# Access and barriers to measures targeted to prevent malaria in pregnancy in rural Kenya*

**DOI:** 10.1111/j.1365-3156.2007.01992.x

**Published:** 2008-02

**Authors:** Priscilla W Gikandi, Abdisalan M Noor, Carol W Gitonga, Antony A Ajanga, Robert W Snow

**Affiliations:** 1 Malaria Public Health and Epidemiology Group, Centre for Geographic Medicine Research-Coast, Kenya Medical Research Institute/Wellcome Trust Research ProgrammeNairobi, Kenya; 2 Centre for Tropical Medicine, University of Oxford, John Radcliffe HospitalOxford, UK

**Keywords:** malaria, pregnancy, antenatal care, intermittent presumptive treatment, sulphadoxine-pyrimethamine, insecticide-treated nets, Kenya

## Abstract

**Objectives:**

To evaluate barriers preventing pregnant women from using insecticide-treated nets (ITN) and intermittent presumptive treatment (IPT) with sulphadoxine-pyrimethamine (SP) 5 years after the launch of the national malaria strategy promoting these measures in Kenya.

**Methods:**

All women aged 15–49 years were interviewed during a community survey in four districts between December 2006 and January 2007. Women pregnant in the last 12 months were asked about their age, parity, education, use of nets, ITN, antenatal care (ANC) services and sulphadoxine-pyrimethamine (SP) (overall and for IPT) during pregnancy. Homestead assets were recorded and used to develop a wealth index. Travel time to ANC clinics was computed using a geographic information system algorithm. Predictors of net and IPT use were defined using multivariate logistic regression.

**Results:**

Overall 68% of pregnant women used a net; 52% used an ITN; 84% attended an ANC clinic at least once and 74% at least twice. Fifty-three percent of women took at least one dose of IPT-SP, however only 22% took two or more doses. Women from the least poor homesteads (OR = 2.53, 1.36–4.68) and those who used IPT services (OR = 1.73, 1.24–2.42) were more likely to sleep under any net. Women who used IPT were more likely to use ITNs (OR = 1.35, 1.03–1.77), while those who lived more than an hour from an ANC clinic were less likely (OR = 0.61, 0.46–0.81) to use ITN. Women with formal education (1.47, 1.01–2.17) and those who used ITN (OR: 1.68, 1.20–2.36) were more likely to have received at least one dose of IPT-SP.

**Conclusion:**

Although the use of ITN had increased 10-fold and the use of IPT fourfold since last measured in 2001, coverage remains low. Provider practices in the delivery of protective measures against malaria must change, supported by community awareness campaigns on the importance of mothers’ use of IPT.

## Introduction

Infection with *Plasmodium falciparum* during pregnancy often results in maternal anaemia and low birth weight, under stable endemic transmission (Brabin 1983; Steketee *et al.* 1996, 2001; Parise *et al.* 1998; Shulman *et al.* 1999). Malaria contributed to an estimated 400 000 cases of anaemia among pregnant African women in 1995 (Guyatt & Snow 2001a) and indirectly caused about 100 000 infant deaths through low birth weight (Guyatt & Snow 2001b). Two relatively simple interventions during pregnancy to reduce the incidence of malaria attributable-anaemia and low birth weight: intermittent presumptive treatment (IPT) using sulphadoxine-pyrimethamine (SP) in the second and third trimesters and the use of insecticide-treated nets (ITN) (Gamble *et al.* 2006; Garner & Gülmezoglu 2006). WHO recommends these two interventions to combat the consequences of malaria in pregnancy by the WHO in the Africa Region (WHO 2004).

In 2000 the Roll Back Malaria (RBM) movement set a target of providing at least 60% of pregnant women with an ITN and at least two courses of IPT by the year 2010 (WHO 2000). Given the relatively high, but variable, uptake of antenatal care (ANC) clinics by pregnant women in Africa (http://www.measuredhs.com), this Abuja target should be attainable by 2010. However there are important differentials in ANC utilization related to parity, age, education and access (Mlay *et al.* 1994; Chandrashekar *et al.* 1998; Sinhababu *et al.* 2006). Understanding the level of coverage of IPT and ITN among women at highest risk of the consequences of malaria on morbidity and pregnancy outcomes is critical to interpretation of overall estimates of RBM coverage targets.

We present the findings of a recent survey of pregnant women and their use of IPT and ITN in four rural districts of Kenya in 2006, 5 years after the launch of the national malaria strategy and we highlight the current barriers to effective coverage among biologically and economically vulnerable women.

## Methods

### The Kenyan malaria in pregnancy strategy

Much of the scientific evidence generated to support the policy position on the use of IPT and ITN during pregnancy in Africa was developed in Kenya (Parise *et al.* 1998; Shulman *et al.* 1999; Van Eijk *et al.* 2002, 2004a,b; Njagi *et al.* 2003; Ter Kuile *et al.* 2003). In April 2001 the Kenyan National Malaria Strategy was launched with the management and prevention of malaria in pregnancy as a major component with a stated aim to ensure that 60% of pregnant women were using an ITN or effective IPT by 2006 (MOH 2001). The Division of Reproductive Health (DRH) of the Kenyan Ministry of Health (MOH) developed an implementation strategy to ensure effective clinical management of anaemia and delivery of IPT among ANC attendees with the support of the John Hopkins Program for International Education in Reproductive Health (JHPIEGO) and financial support from the UK's Department for International Development (DFID). The programme began in July 2000 in the two districts of Kilifi and Busia, as a pilot project and was expanded to four districts (Kwale, Taita-Taveta, Homa Bay and Bondo) in 2002. The fundamentals of the Focussed Antenatal Care programme included the provision of cascade in-service training through decentralized training centres that aimed to reach all cadres of nursing and clinical staff involved in seeing ANC clients. For malaria these in-service training initiatives were supported with laminated visual aids covering the basics of diagnosis and management of anaemia and the timing and dosing of presumptive SP provision during the second and third trimesters of pregnancy. Within the framework of the national malaria strategy, IPT is provided at no charge in public health services.

The provision of ITN to pregnant women formed part of a separate implementation strategy managed by the Division of Malaria Control (DOMC) and other service delivery partners described in detail elsewhere (Noor *et al.* 2007). Briefly, between 2001 and 2004 the predominant source of ITN was the commercial retail sector as part of a DFID-funded programme of social marketing. In 2005 this programme changed to include the delivery of heavily subsidized ITN through maternal and child health and ANC clinics for children aged less than 5 years and pregnant women. In 2006 the DOMC launched a combined programme of ITN distribution with the Kenya Expanded Programme for Immunisation's (KEPI) catch-up mass measles vaccination initiative, providing free ITN to children in 21 districts and free distribution in a further 24 districts not linked to vaccination.

### Study sites and population

The study was conducted in four districts purposively sampled in collaboration with the MOH to provide detailed longitudinal data on changing access to interventions between 2001 and 2006. The study districts represent the range of malaria epidemiological situations that prevail across Kenya: Kwale on the coast with seasonal, high intensity malaria transmission; Bondo on the shores of Lake Victoria with high intensity perennial transmission, Greater Kisii district (combining the new districts of Kisii Central and Gucha) with low, seasonal transmission conditions of the Western highlands, and Makueni district, a semi-arid area with acutely seasonal, low malaria transmission. The use of ANC services, including measures to prevent malaria during pregnancy, at the launch of the KNMS were described by Guyatt *et al.* (2004). Across the four districts in 2001, 11% of rural women pregnant in the last 12 months had slept under a net during the pregnancy and only 4.6% had slept under an ITN, 23% had taken any treatment course of SP during the pregnancy and less than 5.1% of women had had two presumptive treatment courses of SP in their second and third trimesters (Guyatt *et al.* 2004).

Of the 230 rural and urban national census enumeration area (EA) communities sampled during 2001, 72 rural EAs were re-sampled in 2003 to form the basis of a more detailed homestead longitudinal surveillance. Following community sensitization, all homesteads within an EA were mapped using GPS (Garmin *etrex;* Garmin Ltd, Kansas, USA). We explained the purpose of the longitudinal study to heads of homesteads and asked them to participate. In November 2003 all consenting homesteads were recruited into the homestead cohort, *de jure* resident homestead members enumerated including details of date of birth and sex and each homestead member issued a unique identifier linked to their district, EA and household location. Annual censuses were undertaken between December and January 2004/2005 2005/2006 and 2006/2007.

All women aged 15–49 years at the time of the 2006/2007 census were selected from the previous annual census to participate in a detailed interview on their pregnancy histories. Women who provided individual consent for interview and those agreeing to participate were questioned on their birth histories and highest levels of formal education attained. For women reporting a pregnancy that had resulted in a delivery in the last 12 months or who reported being currently pregnant further questions were asked on their use of named ANC services, SP (overall or when they were not sick), net use and whether these were treated in last 6 months or were long-lasting treated nets during the last or current pregnancy.

### Defining wealth quintiles

Details were recorded on each homestead related to key asset indicators including: homestead head education level and occupation, housing characteristics (type of wall, roof and floor), source of drinking water, type of sanitation facility, homestead size and persons per sleeping room. Principal components were used to construct a wealth index (Filmer & Pritchett 2001). Wealth asset indices were developed separately for each district to allow for innate differences in the meaning of different assets between districts. Each homestead was then classified into a district-specific wealth quintile.

### Defining physical access to ANC clinics

Transport routes and topography; government mission and private health services; and physical barriers to travel (hills, rivers and protected areas) were mapped within each district and assembled in arcgis 9.0 (ESRI Inc., USA) (Noor *et al.* 2003). Because most people in the study districts walk to health facilities (Noor *et al.* 2006), walking times were computed using data from the digitized footpaths and roads between the nearest ANC provider and woman's homestead. A travel time algorithm developed in C++ code was used to define speed differentials along the various footpath and road surfaces (Noor *et al.* 2006). Barriers such as rivers, forests and parks were masked as impassable. Where a path traversed a river or other water features, however, travel speed remained unchanged from that of the intersecting road.

### Data entry and analysis

Data entry and storage was undertaken using MS Access (Microsoft, Redmond, USA), analysis was undertaken using STATA version 9.2 (Statacorp 2003, College Station, USA) and arcgis 9.0 (ESRI Inc., USA). A cluster-adjusted chi-squared test was performed to construct precision estimates around proportions and compare them across districts. Regression analyses were undertaken on combined data to examine factors that explained the use of nets; ITN; any IPT and two doses of IPT by pregnant women. Univariate regression analyses were first performed to identify which of the predictor variables were significant to the four outcome measures. In the univariate analyses any predictor with a *P*-value < 0.15 was considered to be a potentially important covariate of the outcome measure. All predictors meeting the entrance criteria were used to estimate a multivariable logistic regression model to identify their combined effect on a given outcome measure. The multivariate models were fitted using the STATA *xtgee* command with an exchangeable working correlation matrix. This procedure uses generalized estimating equations (GEE) to account for the potential correlation of observations on pregnant women seen in the same EA while accounting for the variability between clusters. All results were weighted for unequal probability of selection of EA within each district (weight = 1/probability of selecting an EA). Both the cluster-adjusted chi-squared test and the multivariate regression were adjusted for the effect of the variation between districts. Odds ratio (OR), 95% confidence interval (CI) and *P*-values were recorded for each predictor.

## Results

A total of 4720 eligible women aged 15–49 years were selected for interview, 42 (0.9%) refused participation, 106 (2.2%) normally resident women were visiting at the time of the survey and during subsequent follow-up visits within 2 weeks. Of the 4549 women interviewed, 976 (21.5%) had been pregnant within the last 12 months, including 216 who were pregnant at the time of survey. Overall 81% of pregnant women attended an ANC clinic at least once. ANC usage remained similar and high across all districts with 59% of women averaging at least three visits (Table 1). ANC attendances were higher (92.6% at least once; 85.0% at least twice) when those currently pregnant women, a proportion of whom would be in early pregnancy and would not have began visiting an ANC clinic or using IPT, were excluded from the analysis (Table 1).

**Table 1:** Percentage (cluster adjusted) ANC, any net, ITN and SP use among 976 women seen between December 2006 and January 2007 in 72 rural communities across four districts in Kenya (% (*n*) [95% CI])

	Greater Kisii		Kwale		Bondo		Makueni		Total	
					
	All	Excluding currently pregnant	All	Excluding currently pregnant	All	Excluding currently pregnant	All	Excluding currently pregnant	All	Excluding currently pregnant
Women's characteristics										
Number interviewed	231	185	357	280	213	162	175	133	976	760
Median age (quartiles)	26 (23–31)	27 (23–32)	27 (22–32)	28 (23–32)	25 (23–31)	27 (22–31)	27 (23–32)	28 (23–32)	26 (23–31)	27 (23–32)
ANC use										
Attending ANC at least once	89.2 (206) [84.1–94.3]	95.7 (177) [91.3–100.0]	73.7 (263) [66.1–81.2]	84.6 (237) [77.0–92.3]	80.8 (172) [74.5–87.0]	92.0 (149) [87.1–96.9]	84.6 (148) [79.3–89.9]	94.7 (126) [91.8–97.7]	83.5 (789) [80.4–86.7]	92.6 (689) [90.1–95.1]
Attending ANC at least twice	74.9 (173) [68.2–81.6]	84.3 (156) [76.2–92.4]	68.1 (243) [60.5–75.6]	80.4 (225) [72.8–87.9]	73.7 (157) [67.9–79.6]	88.9 (144) [83.4–94.4]	76 (133) [70.9–81.1]	88.0 (117) [84.4–91.6]	73.6 (706) [70.3–76.9]	85.0 (642) [81.3–88.6]
Attending ANC at least three times	59.7 (138) [53.0–66.4]	69.7 (129) [61.2–78.3]	57.1 (204) [49.4–64.9]	69.3 (194) [61.3–77.3]	60.6 (129) [52.3–68.8]	74.7 (121) [64.5–84.9]	59.4 (104) [53.5–65.4]	74.4 (99) [68.5–80.4]	59.2 (575) [55.7–62.7]	71.5 (543) [67.4–75.6]
Attending ANC at four +times	32 (74) [24.6–39.5]	37.3 (69) [27.9–46.6]	33.9 (121) [27.1–40.7]	41.4 (116) [33.7–49.2]	39.4 (84) [31.4–47.4]	50.0 (81) [41.4–58.6]	32.6 (57) [24.9–40.2]	39.8 (53) [30.9–48.8]	33.5 (336) [29.7–37.3]	40.4 (319) [35.8–45.0]
Use of bed nets										
Using any net during pregnancy	70.6 (163) [63.8–77.3]	68.6 (127) [60.4–76.9]	64.7 (231) [54.7–74.7]	62.9 (176) [49.8–75.9]	78.9 (168) [71.7–86.0]	78.4 (127) [71.4–85.4]	60.6 (106) [49.6–71.5]	54.9 (73) [41.9–67.9]	67.7 (668) [63.3–72.1]	64.9 (503) [59.6–70.4]
Using an ITN during pregnancy	55.8 (129) [47.9–63.8]	54.1 (100) [45.8–62.3]	50.1 (179) [41.5–58.8]	46.1 (129) [34.7–57.4]	58.7 (125) [52.1–65.3]	58.6 (95) [52.2–65.1]	43.4 (76) [33.0–53.8]	36.8 (49) [24.2–49.5]	51.6 (509) [47.3–55.9]	48.4 (373) [43.3–53.5]
SP (any)										
Took any SP	66.0 (153) [58.1–73.8]	69.4 (129) [62.5–76.3]	47.7 (172) [40.6–54.8]	55.2 (153) [47.7–62.8]	48.3 (104) [39.9–56.8]	55.0 (88) [44.0–66.0]	63.4 (111) [53.4–73.4]	71.4 (95) [58.8–84.0]	59.2 (540) [54.8–63.6]	65.1 (463) [60.5–69.7]
At least one dose SP at ANC	64.9 (150) [57.4–72.4]	68.6 (127) [61.7–75.5]	46.5 (166) [40.0–53.0]	54.3 (152) [47.1–61.4]	46.9 (100) [38.6–55.3]	53.1 (86) [42.3–63.9]	62.9 110) [53.0–72.7]	70.7 (94) [58.3–83.0]	58.2 (526) [54.0–62.3]	64.2 (459) [59.7–68.7]
At least two doses SP at ANC	23.8 (55) [16.2–31.4]	24.9 (46) [16.9–32.8]	18.8 (67) [13.0–24.5]	21.1 (59) [15.0–27.2]	16.9 (36) [12.7–21.1]	21.0 (34) [15.7–26.3]	34.3 (60) [26.5–42.1]	38.3 (51) [29.1–47.6]	24.7 (218) [20.8–28.5]	27.1 (190) [22.8–31.4]
At least three doses SP at ANC	5.2 (12) [1.90–8.49]	5.4 (10) [1.5–9.3]	2.0 (7) [7.00–32.0]	2.1 (6) [0.7–3.6]	5.6 (12) [1.88–9.39]	7.4 (12) [2.6–12.2]	9.1 (16) [5.91–12.4]	11.3 (15) [7.7–14.9]	5.6 (47) [4.00–7.22]	6.5 (43) [4.6–8.4]
SP taken when not sick (presumptive)										
At least one dose SP at ANC	58.9 (136) [51.0–66.8]	62.2 (115) [54.8–69.5]	43.9 (157) [37.6–50.4]	51.4 (144) [44.3–58.5]	42.7 (91) [33.9–51.5]	48.1 (78) [36.9–59.4]	56.6 (99) [47.7–64.5]	62.4 (83) [51.9–72.9]	53.0 (483) [49.1–57.0]	58.2 (420) [54.1–62.3]
At least two or more doses SP at ANC	20.8 (48) [12.2–29.3]	21.6 (40) [13.3–30.0]	19.0 (66) [13.2–24.9]	21.4 (60) [15.2–27.7]	13.1 (28) [9.2–17.1]	16.0 (26) [10.8–21.3]	30.3 (53) [23.1–37.5]	33.1 (44) [24.2–41.9]	22.0 (197) [18.0–26.0]	23.9 (170) [19.6–28.2]

### Net and treated net use during pregnancy

Overall 68% of women used a net during their pregnancy with Makueni reporting the lowest coverage (60.6%). Approximately half of the pregnant women (51.6%) used nets treated with insecticide, with small differences between districts and when currently pregnant women were excluded from the analysis (Table 1). Therefore, subsequent results in this section are presented with all women who were pregnant within the last 12 months as the denominator. The routine net distribution through ANC clinics was the main source of nets (53%) followed by the retail commercial sector (22%) while 16% of women slept under nets obtained from a free mass distribution that took place in two phases in July and September (Noor *et al.* 2007) that targeted children under the age of 5 years. The remaining proportion of nets (9%) was obtained through other small-scale distributions (data not shown).

ITN and net use did not vary significantly with the age of the pregnant woman (Table 2). However, when only adolescent mothers (15–19 years of age), who are considered to be a special risk group were selected (*n* = 59), net/ITN use was lower (net use: 56%; ITN use: 44%) than the overall proportion for all other age categories (net use: 69%; ITN use 66%) (data not shown). Although net and ITN use was higher among multigravid women compared to secundigravid and primigravid women this was not statistically significant (net use: Cluster-adjusted *P* = 0.2555; ITN use: Cluster-adjusted *P* = 0.2416) (Table 2). A significantly higher proportion of women with primary or higher level of education used nets (69%) than women without formal education (59%) (*P* = 0.017). 53% of women with some formal education used ITN compared to 49% of women without (*P* = 0.082). The largest disparities in net use were in relation to wealth status (Table 2). Eight-three percent of pregnant women from the least poor homesteads used nets compared to 62% of pregnant women from the poorest homesteads (*P* < 0.001). Similarly, 61% women in the least poor homesteads reported using an ITN compared 49% of those in the poorest homesteads (*P* = 0.082). Distance to ANC clinics, however, influenced both net use (*P* = 0.018) and ITN use (*P* = 0.001), with fewer women reporting use if they lived more than 1 h walking distance to an ANC clinic compared to those within an hour (Table 2).

**Table 2:** Percentage* (cluster adjusted) any net use, ITN and SP use by age, parity, education, wealth and travel times to the ANC clinics in the 72 communities combined across the four districts (% (*n*), [95% CI])

	Net use	ITN use	Presumptive SP 1 + at ANC	Presumptive SP 2 + at ANC
Age*				
15–29 years	68.3 (449) [63.3–73.3]	52.7 (347) [47.9–57.6]	54.1 (327) [49.5–58.6]	23.4 (140) [18.9–25.7]
30–49 years	66.3 (219) [58.9–73.7]	49.4(162) [41.6–57.2]	51.0 (156) [44.1–57.7]	19.2 (57) [12.6–25.7]
Parity				
Primigravid	61.9 (95) [53.7–70.1]	46.5 (69) [37.9–55.1]	53.5 (80) [44.9–62.1]	30.3 (45) [20.8–39.7]
Secundigravid	66.0 (128) [57.1–74.9]	49.3 (96) [39.6–59.0]	58.0 (98) [52.3–63.8]	25.3 (44) [17.4–33.2]
Multigravid	69.9 (445) [64.5–75.2]	53.9 (344) [48.8–59.1]	51.2 (305) [46.4–56.0]	18.4 (108) ‡ [14.0–23.0]
Education				
No formal education	59.3 (127) [52.0–66.7]	45.0 (100) [36.4–53.6]	41.7 (89) [34.0–49.4]	18.9 (35) [11.7–26.2]
Primary or higher education	69.0 (541) ‡ [64.4–73.7]	52.7 (409) † [48.3–57.1]	54.9 (394) ‡ [50.5–59.4]	22.5 (162) [17.9–27.1]
Wealth quintile				
Women residing in most poor homesteads	62.3 (166) [56.2–68.5]	48.9 (129) [42.1–55.8]	47.4 (116) [41.0–53.9]	19.2 (47) [11.8–26.5]
Women residing in very poor homesteads	67.9 (154) [60.1–75.7]	51.7 (118) [42.3–61.0]	46.8 (106) [38.9–54.7]	15.7 (32) [10.3–21.2]
Women residing in poor homesteads	63.2 (136) [53.9–72.5]	44.9 (97) [37.4–52.6]	54.1 (102) [44.8–63.5]	26.0 (50) [19.0–33.0]
Women residing in less poor homesteads	71.9 (117) [62.7–81.2]	57.8 (93) [47.9–67.8]	63.4 (96) [53.2–73.7]	28.8 (45) [20.4–37.1]
Women residing in least poor homesteads	82.8 (94)§ [74.3–91.2]	61.4 (72)† [49.5–73.3]	62.7 (63) ‡ [49.9–75.5]	24.7 (23) [15.7–33.8]
Travel times				
Within 1 h of an ANC clinic	72.8 (338) ‡ [68.0–77.5]	58.2 (254) § [52.5–63.9]	51.2 (265) [45.6–56.7]	20.3 (109) [14.2–26.3]
More than 1 h of an ANC clinic	63.2 (330) [56.5–69.8]	45.9 (265) [40.5–51.3]	54.7 (218) [48.4–61.0]	23.5 (88) [18.2–28.8]

* Age groups were selected pragmatically for sample size reasons rather than targeting special risk groups such as adolescents (15–19 years, *n* = 59).

*Note: P*-value for comparison of proportions between multiple categories of variables:

† *P* < 0.05;

‡ *P* < 0.01;

§ *P* < 0.001.

A cluster adjusted chi-squared test was used to examine the significance of differences in proportions yielding wider confidence intervals and conservative *P*-values compared to the unadjusted chi-squared test. The differences in proportions had a similar pattern when analysis was undertaken excluding women who were currently pregnant (data not shown).

### IPT use during pregnancy

Fifty-nine percent of women reported taking at least one dose of SP from any source during their pregnancy, 53% took at least one dose of SP as IPT and 22% took the recommended two doses or more at an ANC clinic (Table 1). Usage of at least two doses of presumptive IPT at ANC clinics varied between districts (Table 1). When currently pregnant women were excluded from the analysis, 65% of women took at least one dose of SP from any source during pregnancy, 58% took at least one dose as IPT and 24% took the recommended two doses or more (Table 1). Because IPT usage did not vary significantly when the currently pregnant women were excluded from the analysis, subsequent analysis of IPT usage relies on all women who were pregnant within the last 12 months.

There were no significant differences in the proportional use of at least one dose of SP as presumptive IPT from ANC clinics with respect to age, parity or travel time to an ANC clinic (Table 2). Among adolescent mothers (15–19 years), however, IPT usage was lower than that of older women with only 39% and 20% using at least one and two doses of IPT during pregnancy (data not shown). There were also differences with respect to mothers’ education and wealth status: women with no formal education (42%) and those from most poor homesteads (47.4%) were less likely to have used any IPT compared to those with at least primary education (55%) or from the least poor homesteads (62.7%) (*P* = 0.007, and 0.046, respectively). The characteristics of women who had at least two doses of IPT from ANC clinics followed a similar pattern to those who received at least one dose of SP with the exception that more primigravid women (30%) reported accessing at least two doses of SP compared to multigravid women (18%) (*P* = 0.012) (Table 2).

### Multivariate analysis: predictors of net; ITN; and IPT use among pregnant women

When the analysis in Table 2 excluded women who were currently pregnant, the relationship between the explanatory variables and the outcomes (net use; ITN use; and IPT use) remained the same as when all women were included (data not shown). The multivariate analysis was therefore undertaken using data on all women who were pregnant within the last 12 months. Mother's education and age were not statistically associated with the use of nets or ITN when examined in combination with the other predictors. However, the use of nets was significantly associated with (a) the reported use of IPT from an ANC clinic (OR = 1.73; CI = 1.24–2.42); (b) those who were multigravid (OR = 1.53; CI = 1.01–2.35); (c) those who were from the least poor homesteads (OR = 2.53; CI = 1.36–2.09); and (d) those who were within 1 h walking distance to an ANC clinic (OR = 0.67; CI = 0.48–0.93) (Table 3). Use of ITN, however, was significantly associated only with reported use of at least one dose of IPT from an ANC clinic (OR = 1.35; CI = 1.03–1.77) and distance to the ANC clinic used by pregnant women (OR = 0.61; CI = 0.46–0.81).

**Table 3:** Multivariate analysis results of predictors* of net use, ITN use, IPT1 use and IPT2 use among pregnant women in rural Kenya. Data from the four districts were combined for this analysis. OR and 95% CI were adjusted for the effect of districts

	Use of nets by pregnant women		Use of ITN by pregnant women		Use of IPT1 by pregnant women		Use of IPT2 by pregnant women	
				
	OR (95% CI)	*P*-Value	OR (95% CI)	*P*-Value	OR (95% CI)	*P*-Value	OR (95% CI)	*P*-Value
Age category								
15–29 years							Ref.	
30–49 years							0.75 (0.490–1.145)	0.182
Mother's education								
No	Ref.		Ref.		Ref.			
Yes	1.26 (0.86–1.84)	0.230	1.25 (0.85–1.83)	0.250	**1.47 (1.01–2.17)**	**0.049**		
Parity								
Primigravid	Ref.		Ref.					
Secundigravid	1.13 (0.70–1.80)	0.623	1.06 (0.62–1.81)	0.834				
Multigravid	**1.53 (1.01–2.35)**	**0.050**	1.42 (0.93–2.15)	0.103				
Use of IPT								
No	Ref.		Ref.		**NA**	**–**	**NA**	–
Yes	**1.73 (1.24–2.42)**	**0.001**	**1.35 (1.03–1.77)**	**0.032**				
Use of Nets								
No	**NA**	**–**	**NA**	–				
Yes					**1.68 (1.20–2.36)**	**0.003**		
Socio-economic status								
Most poor	Ref.		Ref.		Ref.		Ref.	
Very poor	1.29 (0.88–1.90)	0.192	1.12 (0.73–1.71)	0.602	0.93 (0.61–1.40)	0.718	0.69 (0.42–1.16)	0.162
Poor	0.96 (0.60–1.55)	0.864	0.81 (0.52–1.26)	0.349	1.28 (0.81–2.01)	0.290	1.41 (0.81–2.46)	0.222
Less poor	1.48 (0.94–2.35)	0.094	1.39 (0.88–2.22)	0.169	**1.80 (1.08–2.99)**	**0.024**	1.48 (0.89–2.47)	0.133
Least poor	**2.53 (1.36–4.68)**	**0.003**	1.54 (0.84–2.84)	0.165	1.57 (0.88–2.83)	0.130	1.20 (0.65–2.20)	0.558
Distance to ANC clinics								
Within 1 h	Ref.		Ref.					
Greater than 1 h	**0.67 (0.48–0.93)**	**0.017**	**0.61 (0.46–0.81)**	**0.001**				

* Predictors whose multivariate results are listed in this table had *P* < 0.15 in the univariate analyses for a given outcome measure.

Ref. = reference level. NA =Not applicable: applies when an explanatory variable on a row is the same as the outcome measure on the column indicating that a variable cannot be applied as an outcome measure and as it is own explanatory variable in a regression. Values in bold denote results remained similar when currently pregnant women were excluded from the analysis (data not shown).

For both single and multiple doses of IPT from ANC clinics the multiple regression failed to identify any significant influence on use associated with mothers age, gravidity, wealth status or distance to the nearest ANC clinic (Table 3). However, mothers with primary or higher level of education (OR = 1.47; CI = 1.02–2.17) and those who used a net (OR = 1.68; CI = 1.20–2.34) were more likely to use at least a single dose of IPT from ANC but this was not observed for two or more doses (Table 3).

## Discussion

There have been substantial increases in the use of ITN and at least two doses of presumptive IPT with SP by pregnant women between 2001 (ITN 5% and IPT 5%, Guyatt *et al.* 2004) and 2006 (ITN 52% and IPT 22%, Table 1). These increases can only be attributed to the expansion of national funding and service provision around ITN and IPT delivery. In Bondo district there has been a long-term distribution of nets in Asembo Bay division by the United States Centre for Disease Control and Prevention (CDC) (Philips-Howard *et al.* 2003) as part of community-based studies in the district. However, only two clusters were sampled from this division in which only 17/213 women were seen, of whom 82% used a net and this may partly explain the proportionately higher ITN use in Bondo. More than half of the nets used by pregnant women in all districts were obtained from ANC clinics with an additional 16% being nets that were targeted at children under the age of 5 years through free mass distribution (Noor *et al.* 2007). Information on whether this mother's shared the nets with children under the age of 5 years was not recorded.

A 10- or fourfold increases in coverage over the past 5 years might appear to be great successes, however, both ITN and IPT coverage estimates remain below international and national targets, particularly for IPT. More than 73% of women attended an ANC clinic at least twice during their pregnancy and SP has been widely available at all clinics in these districts between 2005 and 2006 (Zurovac *et al.* 2006). This suggests that there are several barriers to effective implementation of IPT and we would argue that these are probably factors related to access to services; socio-economic status and factors not reported here but operating at the provider or facility level. We did not examine provider awareness or perceptions about the provision of SP to pregnant women as IPT and therefore cannot comment directly on this. Interestingly, of all pregnant women who used nets, 53.4% had obtained them through routine services at clinics targeting pregnant women and children under the age of 5 years (data not shown). Of these, 58% had at least one dose of IPT-SP; only 24% had two or more doses. This suggests three probable reasons for low IPT uptake: health workers at clinics that provide IPT services do not give appropriate advice such that many women do not return for follow-up doses; or where appropriate advice is provided, many women do not return for follow-up IPT doses due to personal, geographic and/or socio-economic factors; or many clinics, including those that distribute nets, do not provide IPT services at all.

Indirectly, SP as an effective treatment for malaria was increasingly less well accepted from both observed clinical efficacy (EANMAT 2003) and observed prescriptions at clinics well before the introduction of the new drug policy involving artemether-lumefantrine to clinics during the third quarter of 2006 (Amin *et al.* 2007). Despite declining clinical efficacy of SP this drug might still offer some protection against placental infections and the adverse consequences of malaria during pregnancy in Kenyan women (Van Eijk *et al.* 2007). At least three doses of SP might be a better strategy for IPT during pregnancy where HIV prevalence is high (Parise *et al.* 1998). In our study only 6% of women accessed at least three doses (Table 1). The continued use of SP as IPT for pregnant women is a difficult message to convey to providers of ANC services who are now told not to prescribe SP for clinical malaria. Removing SP from treatment kits for public health facilities over the next 12 months will create a vacuum with respect to IPT for pregnant women at the clinic level. Further studies into the impact of the removal of SP from the treatment kit, IPT provider practices at ANC clinics and the level of awareness and perception of mothers with regards to ITN/IPT usage are required to confirm the results of this study for informed decision-making.

In Kenya, despite the low IPT coverage, high ANC usage has consistently been reported and in the study districts usage is over 80% for at least one visit to ANC clinic and 74% for at least two visits during pregnancy (Table 2). Therefore, we would suggest that changing provider practices at ANC clinics in the delivery of IPT services, supported by community awareness campaigns to educate mothers on the importance of IPT usage, is required. Any new IPT strategies will have to consider the increasing malaria parasite resistance to SP and explore other candidate therapies, probably as combination regimens. Several such candidates are in phase II/III clinical trials, some with good prospects for the near future (Vallely *et al.* 2007).
